# Fusion of EEG and EMG signals for detecting pre-movement intention of sitting and standing in healthy individuals and patients with spinal cord injury

**DOI:** 10.3389/fnins.2025.1532099

**Published:** 2025-01-24

**Authors:** Chenyang Li, Yuchen Xu, Tao Feng, Minmin Wang, Xiaomei Zhang, Li Zhang, Ruidong Cheng, Weihai Chen, Weidong Chen, Shaomin Zhang

**Affiliations:** ^1^Key Laboratory of Biomedical Engineering of Ministry of Education, Qiushi Academy for Advanced Studies, Zhejiang University, Hangzhou, China; ^2^Department of Biomedical Engineering, Zhejiang University, Hangzhou, China; ^3^Center of Excellence in Biomedical Research on Advanced Integrated-on-Chips Neurotechnologies (CenBRAIN Neurotech), School of Engineering, Westlake University, Hangzhou, China; ^4^Westlake Institute for Optoelectronics, Westlake University, Hangzhou, China; ^5^The First Affiliated Hospital, Zhejiang University School of Medicine, Hangzhou, China; ^6^Department of Rehabilitation Medicine, Center for Rehabilitation Medicine, Zhejiang Provincial People's Hospital (Affiliated People's Hospital Hangzhou Medical College), Hangzhou, China; ^7^School of Automation Science and Electrical Engineering, Beihang University, Beijing, China; ^8^Hangzhou Innovation Institute, Beihang University, Hangzhou, Zhejiang, China; ^9^State Key Laboratory of Brain-Machine Intelligence, Zhejiang University, Hangzhou, China; ^10^The MOE Frontier Science Center for Brain Science & Brain-machine Integration, Zhejiang University, Hangzhou, China

**Keywords:** multimodal, human-machine interface, electroencephalography, surface electromyography, muscular fatigue, pre-movement intention detection

## Abstract

**Introduction:**

Rehabilitation devices assist individuals with movement disorders by supporting daily activities and facilitating effective rehabilitation training. Accurate and early motor intention detection is vital for real-time device applications. However, traditional methods of motor intention detection often rely on single-mode signals, such as EEG or EMG alone, which can be limited by low signal quality and reduced stability. This study proposes a multimodal fusion method based on EEG–EMG functional connectivity to detect sitting and standing intentions before movement execution, enabling timely intervention and reducing latency in rehabilitation devices.

**Methods:**

Eight healthy subjects and five spinal cord injury (SCI) patients performed cue-based sit-to-stand and stand-to-sit transition tasks while EEG and EMG data were recorded simultaneously. We constructed EEG–EMG functional connectivity networks using data epochs from the 1.5-s period prior to movement onset. Pairwise spatial filters were then designed to extract discriminative spatial network topologies. Each filter paired with a support vector machine classifier to classify future movements into one of three classes: sit-to-stand, stand-to-sit, or rest. The final prediction was determined using a majority voting scheme.

**Results:**

Among the three functional connectivity methods investigated—coherence, Pearson correlation coefficient and mutual information (MI)—the MI-based EEG–EMG network showed the highest decoding performance (94.33%), outperforming both EEG (73.89%) and EMG (89.16%). The robustness of the fusion method was further validated through a fatigue training experiment with healthy subjects. The fusion method achieved 92.87% accuracy during the post-fatigue stage, with no significant difference compared to the pre-fatigue stage (*p* > 0.05). Additionally, the proposed method using pre-movement windows achieved accuracy comparable to trans-movement windows (*p* > 0.05 for both pre- and post-fatigue stages). For the SCI patients, the fusion method showed improved accuracy, achieving 87.54% compared to single- modality methods (EEG: 83.03%, EMG: 84.13%), suggesting that the fusion method could be promising for practical rehabilitation applications.

**Conclusion:**

Our results demonstrated that the proposed multimodal fusion method significantly enhances the performance of detecting human motor intentions. By enabling early detection of sitting and standing intentions, this method holds the potential to offer more accurate and timely interventions within rehabilitation systems.

## 1 Introduction

Functional movement disorders resulting from brain or spinal cord lesions often lead to loss of independence in daily activities and significantly impact patients’ quality of life (Langhorne et al., 2009; Savic et al., 2018). A key focus of rehabilitation for individuals with SCI or other neurological impairments is the restoration of basic motor functions, such as sitting and standing, which are fundamental functional movement in daily life.

Robotic-assisted rehabilitation devices have demonstrated promising potential for enhancing functional recovery of movement, compared to traditional methods (Nam et al., 2017; Carpino et al., 2018). These robotic systems can promote recovery of movement and facilitate neuroplasticity through intensive and repetitive motor training (Edgerton and Roy, 2009; Mekki et al., 2018). Compared to passive movement, active engagement of patients leads to more substantial motor function improvements (Hogan et al., 2006; Hu et al., 2009; Krebs et al., 2009). Human–machine interfaces (HMIs) are widely used for the active control of rehabilitation devices (Esposito et al., 2021). In HMI systems, accurate and early decoding of motor intentions is crucial for real-time applications, allowing robotic devices to respond promptly to the patient’s voluntary movements. This would enable more proactive assistance for individuals with spinal cord injuries or other movement impairments, ultimately improving rehabilitation outcomes. However, only a few studies have focused on the early detection of movement intentions in the context of lower limb function (Shafiul Hasan et al., 2020).

Over the recent decades, bioelectrical signals, force, and velocity have been used to detect human movement intentions (Blank et al., 2014). For bioelectrical signals, electroencephalogram (EEG) and surface electromyogram (EMG) are commonly employed for controlling rehabilitation and assistive robotic devices (Ang and Guan, 2013; Leonardis et al., 2015; Lin et al., 2023). Pre-movement brain activity changes in the EEG signals have been used to detect movement intentions for tasks such as sitting and standing (Bulea et al., 2014; Li C. et al., 2023), walking (Sburlea et al., 2015), wrist extensions (Bai et al., 2011) and finger movements (Wang et al., 2020). EMG signal changes occur approximately 20–150 ms prior to muscle contraction (Norman and Komi, 1979; Lu et al., 2024), which has been shown to reliably predict movement intention before the onset of physical movement (Kirchner et al., 2014; Trigili et al., 2019). In contrast, methods based on force and velocity information inevitably limited by time delays, as these signals can only be measured after the initiation of limb movement.

However, relying solely on single-modality EEG or EMG cannot fully meet the requirements for effective active control. For instance, muscle fatigue from repetitive contractions and insufficient residual myoelectric activity in patients can reduce the accuracy of motion intentions decoding using EMG (Enoka and Duchateau, 2008; Cipriani et al., 2011). While EEG signals have a lower amplitude and are prone to interference from noise, such as environmental artifacts or non-motor-related brain activity, making them less accurate and reliable (Leeb et al., 2011; Li et al., 2017). Fusing multimodal bio-signals show potential in leveraging complementary information and providing a more accurate and comprehensive description of user’s movement intention, thus improving the recognition efficiency. Recent research studies have proposed approaches for the fusion of EEG and EMG to recognize upper/lower limb motion. Jacob Tryon et al. proposed an EEG–EMG fusion method for classification using a Weighted Average fusion method (Tryon and Trejos, 2020). A Convolutional Neural Network (CNN) model based on EEG–EMG fusion was developed to classify task weight during dynamic elbow flexion–extension movements (Tryon and Trejos, 2021). Yang et al. proposed a fusion approach using the functional connectivity and graph convolutional network, improving the accuracy and reliability of hand motion recognition (Yang et al., 2022). Al-Quraishi et al. developed a technique for fusion of EEG and EMG based on discriminant correlation analysis combined with different classification models for identifying bilateral ankle joint movements (Al-Quraishi et al., 2021). Chowdhury et al. used band-limited power time-courses (CBPT) to extract the cortico-muscular feature associated with EEG and EMG to classify hand grasp movements (Chowdhury et al., 2019). Jiang et al. present a multimodal EEG–EMG fusion network E^2^FNet for various hand motor intent recognition (Jiang et al., 2024). A classifier combing EEG and EMG features was implemented to detect upper limb movement intentions in chronic stroke patients, with the goal of controlling wearable robotic systems (Jo et al., 2022). Li et al. proposed a hybrid EEG–EMG movement recognition method that employs a sequential learning model incorporating a Graph Isomorphic Network to process a sequence of graph-structured data derived from EEG and EMG signals (Li H. et al., 2023). Combined temporal and spectral features of EEG and time domain features of EMG could improve single-trial movement classification in SCI patients with residual EMG, which indicated the feasibility and usability of using HMI system for motor rehabilitation of patients with SCI (Leerskov et al., 2020). However, these current fusion strategies have some shortcomings in terms of practical applications of robotic rehabilitation systems. First, the EEG–EMG functional coupling information is often ignored, while the functional coupling is essential for movement preparation and execution. Given that EEG and EMG signals are nonlinear and complex (Stam, 2005), more suitable functional coupling methods should be investigated. Second, the feasibility of using pre-movement windows for classification tasks in the control of assistive and rehabilitation devices needs further assessment, as decoding movement intention in pre-movement state enables parallel control rehabilitation devices alongside real movement execution. Third, the robustness of the fusion method against the muscular fatigue should be evaluated. Finally, the feasibility to detect the intention based on the fusion of EEG and EMG for the SCI patients should be explored to demonstrate its potential for clinical applications.

This study proposed a multimodal fusion method for detecting sitting and standing intentions based on EEG–EMG functional connectivity, utilizing signals recorded prior to movement execution. A supervised learning model was developed to extract discriminative spatial patterns from EEG–EMG functional connectivity networks. Various connectivity methods, including coherence (COH) (Zhang et al., 2023), correlation coefficient (CC) (Chowdhury et al., 2019), and mutual information (MI) (Kim et al., 2017), were investigated to identify the approach that offers the best performance for detecting sitting and standing movement intentions. The feasibility of utilizing time windows prior to movement onset for intention detection was examined by evaluating classification accuracy across different durations, including 2, 1.5, 1, 0.75, and 0.5 s. In addition, we compared the performance of pre-movement windows with trans-movement and ongoing movement windows to demonstrate that reliable intention detection can be achieved prior to the movement onset. To assess the practical reliability of the proposed approach, its robustness was evaluated under muscular fatigue conditions. Preliminary tests were also conducted on SCI patients to validate its potential in clinical settings. This method aimed to improve the accuracy and responsiveness of assistive and rehabilitation devices, and provided a promising solution for real-time intention detection.

## 2 Materials and methods

### 2.1 Subjects and experimental paradigm

We recruited 8 healthy subjects (2 female, 6 males; 21–27 years old) without any lower limb pathology or neurological abnormalities and 5 SCI patients (1 female, 4 males; 24–39 years old) in this study. The demographic and clinical characteristics of SCI patients are reported in Table 1. All participants were informed about the experimental procedure and sign the consent form before the experiment. The studies involving humans were approved by Zhejiang Provincial People’s Hospital. The studies were conducted in accordance with the local legislation and institutional requirements.

**Table 1:** Demographic and clinical characteristics of SCI patients.

Patient	Age	Sex	Injury level	ASIA	Time since injury (months)
P1	29	male	L2	D	17
P2	37	female	C4	D	7
P3	24	male	L4	C	5
P4	39	male	C6	D	5
P5	34	male	C5	D	4

**Figure 1:**
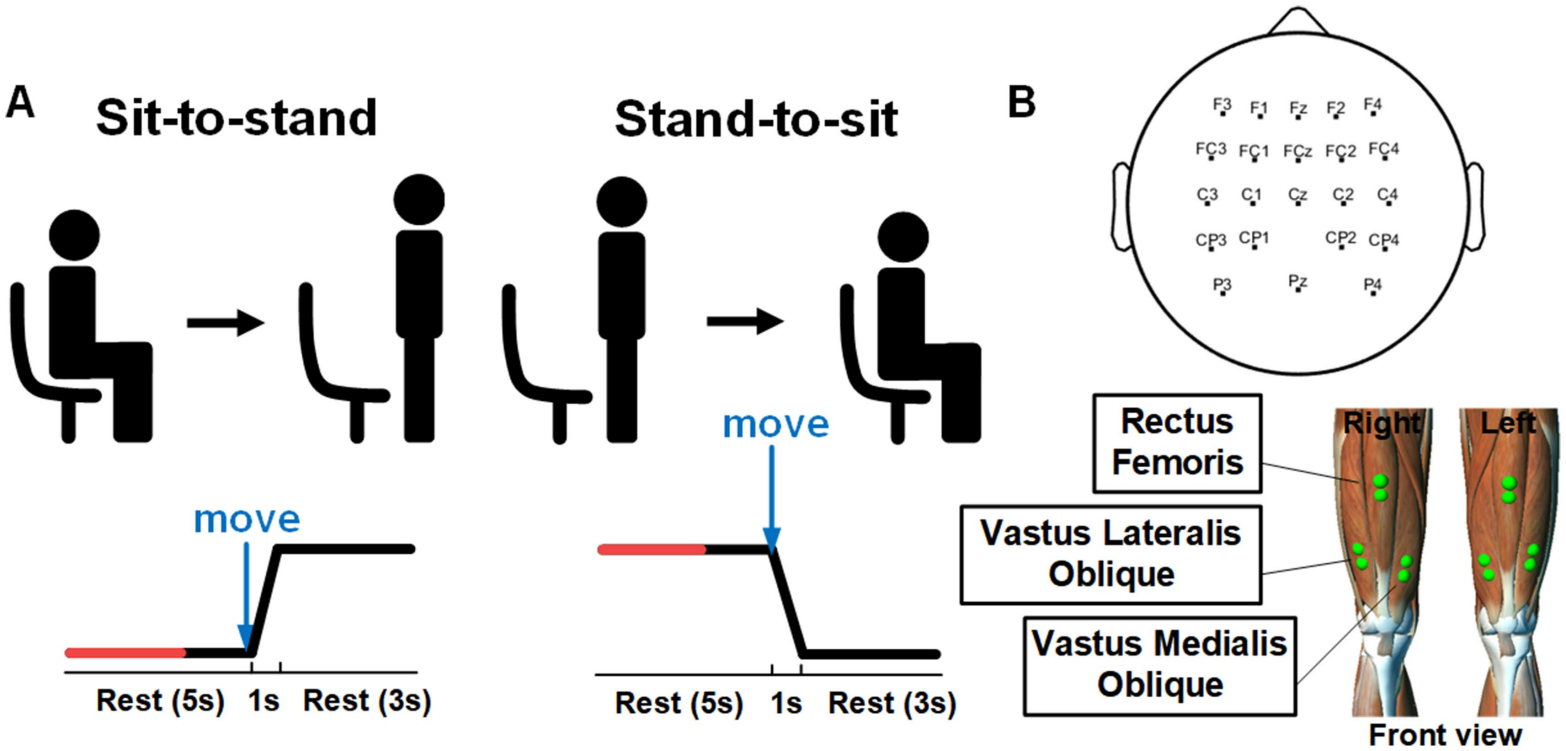
**(A)** Experimental paradigm and visual cues for sit-to-stand and stand-to-sit transitions within a single trial; reprinted with permission from Li C. et al. (2023). Copyright 2023, IEEE. **(B)** Electrode positions for recording EEG and EMG signals.

The experiments involving both healthy subjects and SCI patients were conducted in quiet and controlled environments. For the healthy subjects, EEG and EMG signals were recorded in a laboratory environment with soft non-fluorescent lighting in the room to prevent any flicker or light interference that could affect the EEG signals. The experiments with SCI patients were performed in a controlled environment at a local hospital, under the supervision of specialized medical staff. A visual cue-based paradigm was used in this study, as shown in Figure 1A. Each trial started with the subjects sitting in the chair for 5 s. They were asked to initiate sit-to-stand when the progress bar advanced to the specified position (“move”), and then maintained the current state for 3 s. Afterwards, the progress bar updated and gave similar visual cue to initiate the transition of stand-to-sit. Thus, each trial consisted of one sit-to-stand and one stand-to-sit transition. For the healthy subjects, the task consisted of three procedures, the first of which was a pre-fatigue stage, followed by a fatigue training procedure and a post-fatigue stage. For both pre-fatigue and post-fatigue procedures, each procedure includes 40 trials. During fatigue training period, each subject performed continuous sit-to-stand and stand-to-sit exercises. This process was repeated for 3 min, or until the subjects was exhausted (Al-Quraishi et al., 2021). For the SCI patients, each patient completed a total of 40 trials, which included 40 sit-to-stand and 40 stand-to-sit transitions.

### 2.2 Data acquisition and pre-processing

EEG and EMG data were acquired simultaneously. The EEG signals were recorded using active Ag/AgCl electrodes (64 Channels, NeuSen W) with 22 channels (FZ, F1-F4, FCZ, FC1-FC4, CZ, C1-C4, CP1-CP4, PZ, P3, P4) placed on the scalp according to the 10–20 system (see Figure 1B). The recorded signals were sampled at 1 kHz and referenced to the channel CPz. The channel AFz was used as the ground. The impedance of all selected electrodes was reduced to 5 kΩ. The raw recorded EEG data were bandpass filtered from 0.5 to 45 Hz by utilizing a zero-phase fourth-order Butterworth filter. The common average reference (CAR) was conducted to re-referenced EEG signals to eliminate the global background activity. Afterward, we performed Infomax independent component analysis (ICA) using the EEGLAB toolbox (Delorme and Makeig, 2004). The resulting independent components (ICs) were classified with the ICLabel algorithm into brain and artifactual categories (Pion-Tonachini et al., 2019). In this study, ICs with probabilities for the artifactual classes (“Muscle,” “Eye,” “Heart”) were higher than 70% were labeled as artifacts and removed. The cleaned EEG signals were then reconstructed by back-projecting the remaining components into the original electrode space.

The EMG signals were recorded at 1.5 kHz with a wireless Noraxon Desktop DTS system (Noraxon, USA). The EMG signals from bilateral Rectus Femoris (RF), Vastus Lateralis Oblique (VLO) and Vastus Medialis Oblique (VMO) muscles were recorded with 6 channels. The raw EMG signals were bandpass filtered of 15–300 Hz and notch filtered of 48–52 Hz by using a fourth-order Butterworth filter. Afterward, the Teager-Kaiser energy operator (Solnik et al., 2008) was applied to detect actual movement onset from EMG recordings. The threshold T was set as T = *μ* + h*σ, where μ and σ were the mean and standard deviation of the baseline signal (from −2 to −3 s prior the onset of visual cue), and h was set to 5 empirically. If there were more than 20 consecutive points that exceeded T, we identified the first sample as the actual movement onset.

The pre-processed EEG and EMG signals were segmented to 6-s trials (−4 to 2 s from the movement onset). Based on the previous study (Bulea et al., 2014) that used a 1.5-s window prior to movement onset for intention detection, we defined the “intention” period as [−1.5 0] s for initial analysis. The “rest” period was set as the 1.5-s window from [−4–2.5] s. Time 0 s corresponds to the actual movement onset. To further investigate the impact of window length, we compared various window lengths prior to movement onset (2, 1.5, 1, 0.75, and 0.5 s). For each window, we obtained 22-channel EEG data, 6-channel EMG data and 28-channel EEG–EMG data.

### 2.3 Functional connectivity analysis

After preprocessing, the functional connectivity values of each of the two channels are computed. When constructing the networks, three approaches of functional connectivity are employed to represent the couplings of EEG–EMG, EEG–EEG and EMG–EMG. We calculate the COH, CC and MI between pairwise electrodes, and they are computed by (1–3), respectively:


(1) $$C_{xy}=|P_{xy}{|}^{2}/\left(P_{xx}\cdot P_{yy}\right)$$

where $C_{xy}$ is the coherence of x and y, $P_{xy}$ is the cross-spectral density of the paired signals x and y, $P_{xx}$ and $P_{yy}$ are the respective auto-spectral densities. COH within 13–30 Hz is set as the edge weight in this study;


(2) $$r_{xy}=cov\left(x,y\right)/\left(\sigma_{x}\cdot \sigma_{y}\right)$$

where $r_{xy}$ is the Pearson coefficient, $cov\left(x,y\right)$ is the covariance between the paired signals x and y, $\sigma_{x}$ and $\sigma_{y}$ are the standard deviations of x and y;


(3) $$MI_{xy}=\underset{x}{\sum }\underset{y}{\sum }\left(\frac{\rho \left(x,y\right)}{\rho \left(x\right)\rho \left(y\right)}\right)$$

where $MI_{xy}$ is the mutual information between x and y, $\rho \left(x,y\right)$ means the joint probability distribution of x and y, and $\rho \left(x\right)$ and $\rho \left(y\right)$ are the probability distributions of x and y, respectively. For the CC and MI calculations, we utilized the broadband frequency range of the preprocessed EEG and EMG signals.

For each trial window, 22*22 EEG–EEG, 6*6 EMG–EMG, and 28*28 EEG–EMG adjacency matrices were constructed using the three different measurement methods mentioned above, respectively. For each network, the adjacency matrix is standardized by min-max standardization. Then, we applied multi-class discriminative spatial network pattern features procedure to extract spatial filters and features (Li et al., 2024).

### 2.4 Features extraction and classification

A supervised learning model was developed to identify sitting and standing intentions. The goal of the model is to determine the optimal projection to maximize the difference by maximizing the variance in the network of one movement while minimizing the variance in the network of another movement. We designed spatial filters that maximizing the variance of one class while minimizing the variance of another class. Specifically, $M_{i}$ denotes the network matrix of class i , and $M_{j}$ denotes the network matrix of class j . Their spatial covariance matrix is defined as (4) and (5):


(4) $$C_{i}=\frac{1}{N_{i}}\underset{N_{i}}{\sum }M_{i}M_{i}^{T}$$

(5) $$C_{j}=\frac{1}{N_{j}}\underset{j}{\sum }M_{j}M_{j}^{T}$$

where $N_{i}$ and $N_{j}$ are the number of trials of class i and class j , respectively. Spatial filters are selected to maximize the ratio of the transformed data variance between two classes, as shown in (6):


(6) $$\arg\max_{w}J\left(w\right)=\frac{w^{T}C_{i}w}{w^{T}C_{j}w}\;s.t.\;||w^{T}C_{j}w|{|}_{2}\;=1$$

where w is the spatial filter and $||\cdot |{|}_{2}$ is the $L_{2}$ -norm. We used the method of Lagrange multipliers, and reformulated the constrained optimization problem:


(7) $$L\left(w,\lambda \right)=w^{T}C_{i}w-\lambda \left(w^{T}C_{j}w-1\right)$$

where λ represents the Lagrange multiplier. To find the optimal w and λ , we take the derivative of (7) with respect to w and λ , and set them to 0, as shown in (8):


(8) $$\frac{\partial L}{\partial w}=0,\frac{\partial L}{\partial \lambda }=0$$

For this optimization problem, we solve the generalized eigenvalue problem (9):


(9) $$C_{i}w=\lambda C_{j}w$$

where λ represent the eigenvalue of the generalized eigenvalue equation and w is the corresponding eigenvector. The solution for multiple spatial filters involves (10):


(10) $${\left(C_{j}\right)}^{-1}C_{i}W=\sum W$$

where W represents the spatial filters, and ∑ is the eigenvalue diagonal matrix. We selected the 2 largest and 2 smallest generalized eigenvalues to obtain a set of the most discriminative spatial filters. Support vector machine (SVM) of linear kernel is used for the classification and 10-fold cross-validations was employed to evaluate the robustness of our classification model.

**Figure 2:**
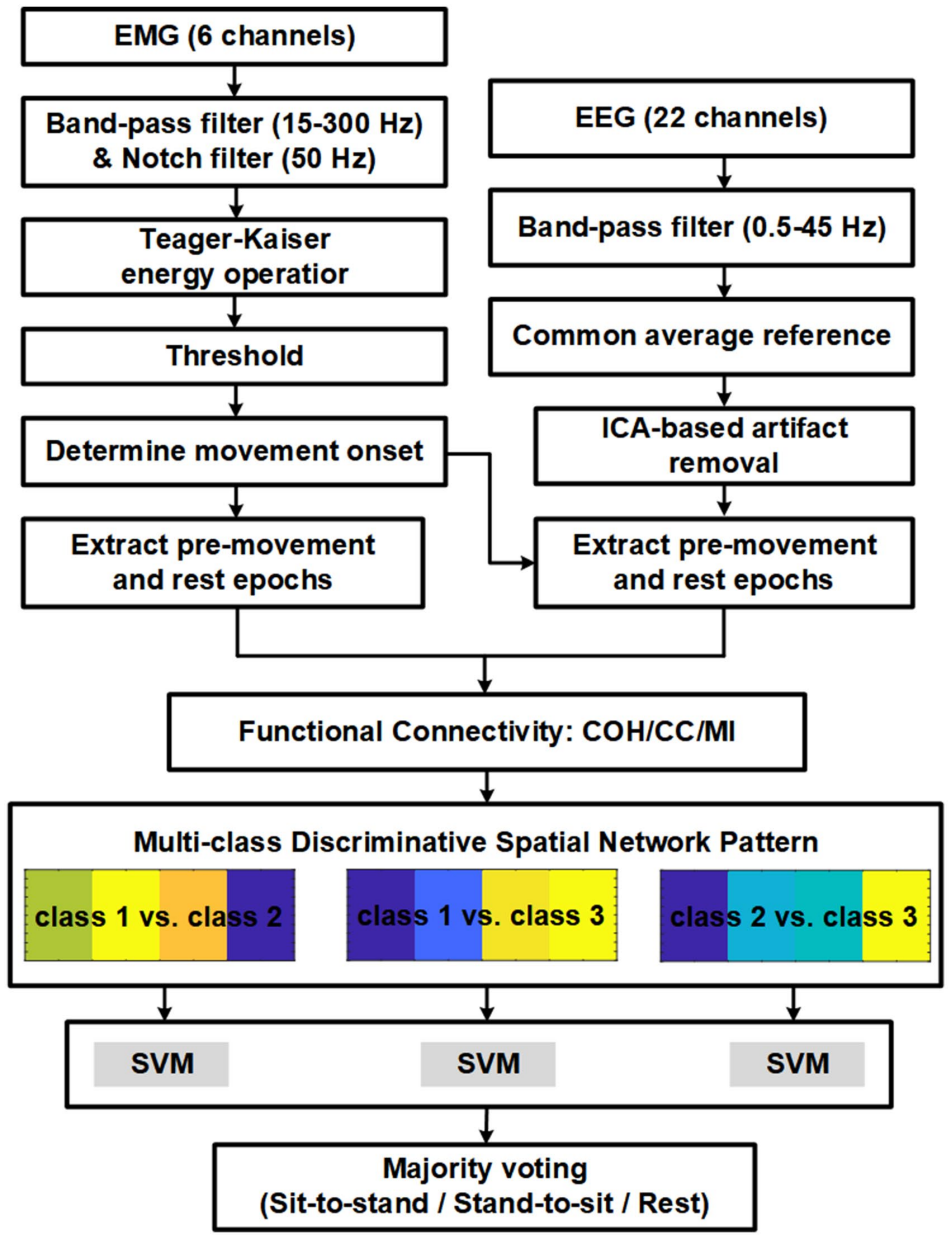
Schematic representation of the proposed multimodal fusion method.

To extract multi-class discriminative spatial network pattern features and classifying three classes, we used “Pair-Wise” based approach that involves constructing and training 3*(3–1)/2 = 3 spatial filters and binary classifiers for each pair of classes. During the testing phase, each test sample is projected through the spatial filters associated with each pair of classes, resulting in features that then input into the corresponding binary classifier. The final predicted class is determined by aggregating the results from all pairwise classifications using a majority voting scheme, where the class with the most votes is selected as the final prediction. In this study, we use the MATLAB (Mathworks Inc., MA, USA) function binoinv to compute the significant chance level $T=\mathrm{binoinv}\left(1-\alpha ,n,1/c\right)\times 100/n$ , where α = 0.05 is the significance level, *n* = 160 is the number of samples for subject, c = 3 is the number of classes. In this study, the significant chance level for classification was determined as 39.375%. Schematic representation of the proposed multimodal fusion method is shown in Figure 2.

## 3 Results

### 3.1 Impact of fatigue training on MNF and MDF of EMG

Frequency-domain features are widely used to evaluate muscle fatigue, with mean frequency (MNF) and median frequency (MDF) regarded as standard indicators for examining the impact of muscle fatigue on EMG signals (Moritani et al., 1982; Al-Quraishi et al., 2021). Muscle fatigue is typically characterized by a downward shift in the EMG frequency spectrum. In this study, we used MNF and MDF to analyze fatigue effects in the thigh muscles, as defined in (11) and (12),


(11) $$MNF=\overset{M}{\underset{i=1}{\sum }}f_{i}P_{i}/\overset{M}{\underset{i=1}{\sum }}P_{i}$$

(12) $$\overset{MDF}{\underset{i=1}{\sum }}P_{i}=\overset{M}{\underset{i=MDF}{\sum }}P_{i}=\frac{1}{2}\overset{M}{\underset{i=1}{\sum }}P_{i}$$

where $f_{i}$ is a frequency value at a frequency bin i , $P_{i}$ represents the EMG power spectrum at a frequency bin i and M is the length of frequency bin.

**Figure 3:**
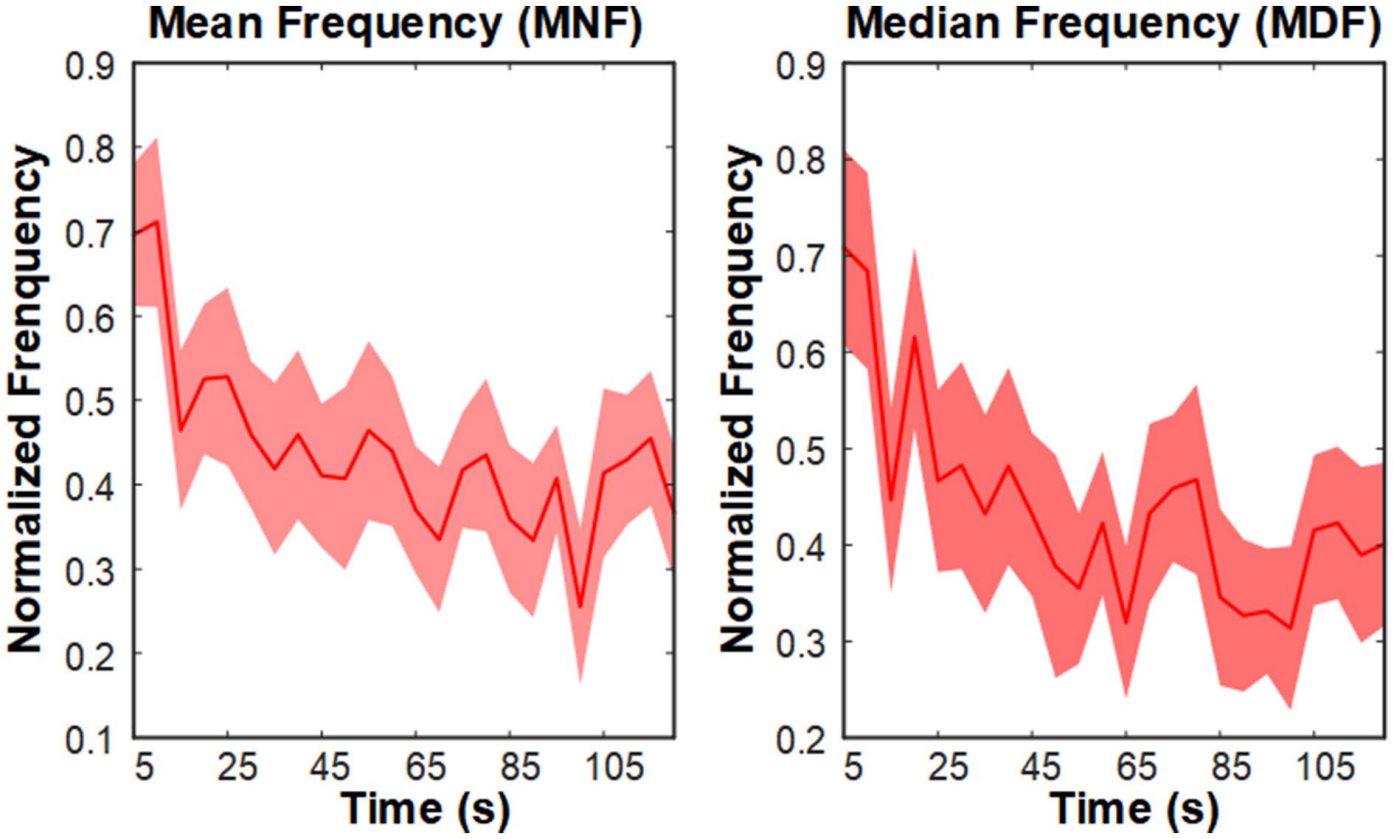
Grand average MNF and MDF trends of the right TA muscle during the fatigue training phase.

Figure 3 shows the trends of MNF and MDF for a representative muscle (RF-right), both of which exhibited a progressive decline over time. These findings highlight the pronounced effect of fatigue training, consistent with established patterns observed in fatigued muscles.

### 3.2 Comparison of the decoding accuracy of different functional connectivity methods

Based on the previous study on decoding sitting and standing intentions using data from 1.5 s before movement onset up to movement onset (Bulea et al., 2014), we initially analyzed data epochs 1.5 s before movement onset to examine the effects of different functional connectivity approaches and modalities on the intention detection. We compared the performance of three functional connectivity metrics (COH, CC, and MI) across three modalities (EEG–EMG, EEG-only, and EMG-only). The classification accuracies of the three connectivity approaches under three modalities for all the subjects during the pre-fatigue stage are shown in Table 2. The results demonstrate that the classification of the EEG–EMG network weighted by MI achieved highest accuracy (94.33%).

**Table 2:** The classification accuracies of healthy subjects for different functional connectivity methods across three modalities during the pre-fatigue stage.

Subjects	Decoding accuracy % (pre-fatigue)								
	EEG–EMG			EEG			EMG		
	COH	CC	MI	COH	CC	MI	COH	CC	MI
S1	82.5	84.38	95	36.88	85	66.25	81.88	83.75	94.38
S2	74.38	89.38	97.5	52.5	89.38	80	79.38	78.13	91.25
S3	75.63	90	96.25	58.75	90	82.5	66.88	77.5	85.63
S4	76.88	71.25	95.63	53.75	53.75	60.63	65.63	85.63	96.25
S5	65	86.25	96.25	41.25	85.63	83.13	65	76.25	93.13
S6	81.88	96.25	86.88	45.63	88.75	85	97.5	96.25	82.5
S7	60.63	77.5	95.63	38.13	80	63.13	58.75	83.13	83.13
S8	64.79	77.45	91.53	46.88	71.48	70.52	69.88	81.36	87.06
Mean	72.71	84.06	94.33	46.72	80.5	73.90	73.11	82.75	89.16
Std	8.25	8.18	3.48	7.83	12.43	9.87	12.47	6.38	5.28

**Figure 4:**
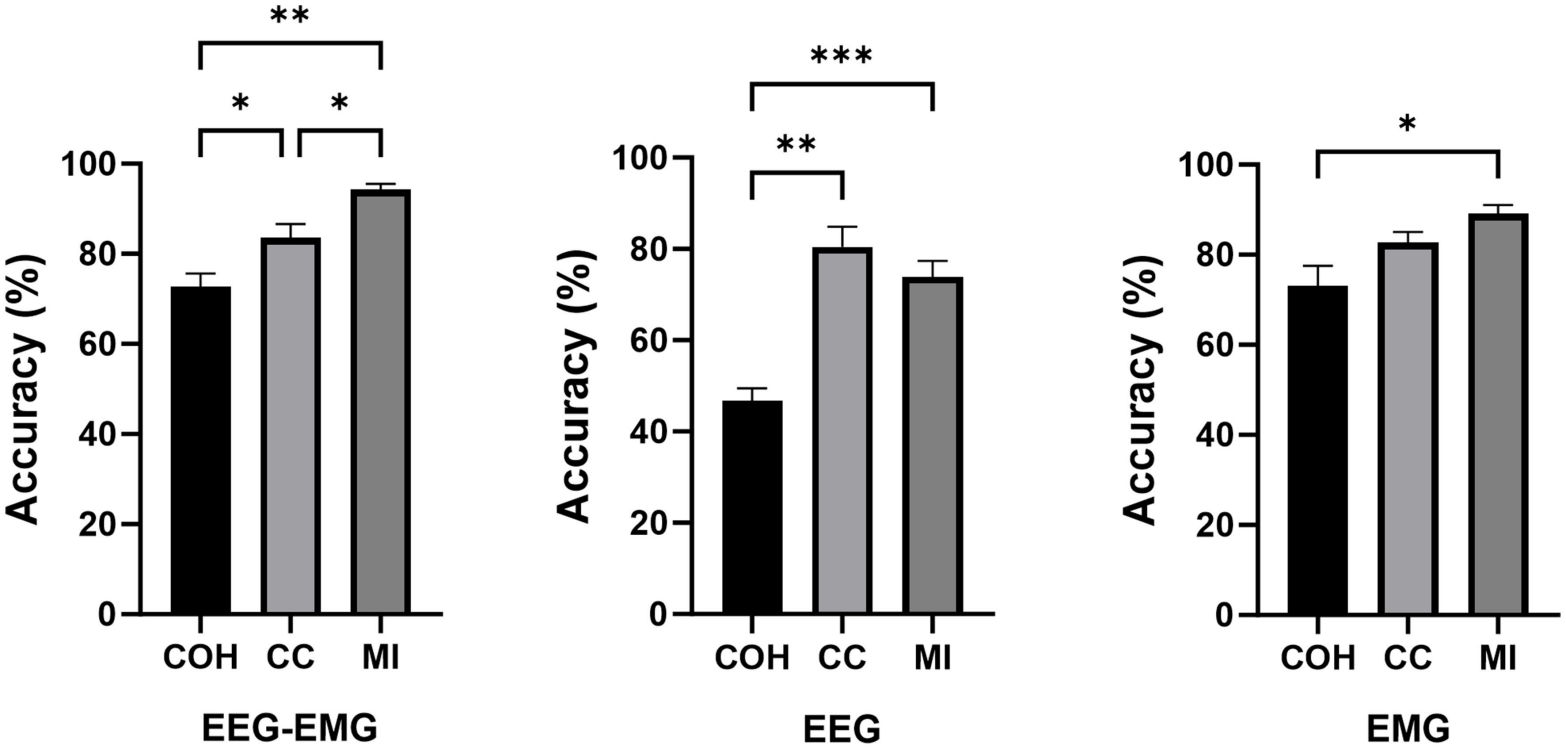
Comparison of classification accuracies across different connectivity methods during the pre-fatigue stage (*p < 0.05, **p < 0.01, ***p < 0.001).

A one-way repeated measures analysis of variance (ANOVA) was used to evaluate the significance of differences among the connectivity methods. As shown in Figure 4, the ANOVA analysis results showed that the decoding accuracy varied significantly across connectivity methods for all three modalities (EEG–EMG: *p* = 8.956e-05; EEG: *p* = 0.0001; EMG: *p* = 0.01). Specifically, for the EEG–EMG network, the accuracies were 72.71, 84.06 and 94.33% for COH, CC and MI, respectively. Post-hoc comparisons test revealed significant differences between COH and CC (*p* = 0.0244), COH and MI (*p* = 0.0011), and CC and MI (*p* = 0.0469). For the EEG network, the accuracies were 46.72, 80.5 and 73.89% for COH, CC and MI, respectively. There were significant differences between COH and CC (*p* = 0.0012), and COH and MI (*p* = 4.7143e-4), while there was no statistical difference between CC and MI (*p* = 0.1371). For the EMG network, the accuracies were 73.11, 82.75, and 89.16% for COH, CC and MI, respectively. There was only a significant difference between COH and MI (*p* = 0.0365), while no significant difference was observed between COH and CC (*p* = 0.0549), and CC and MI (*p* = 0.2095). These findings suggest that the MI functional connectivity method provides robust performance across both unimodal (EEG, EMG) and multimodal (EEG–EMG) settings, particularly in the EEG–EMG network. This underscores MI as the most effective functional connectivity method for decoding intentions in our study.

**Figure 5:**
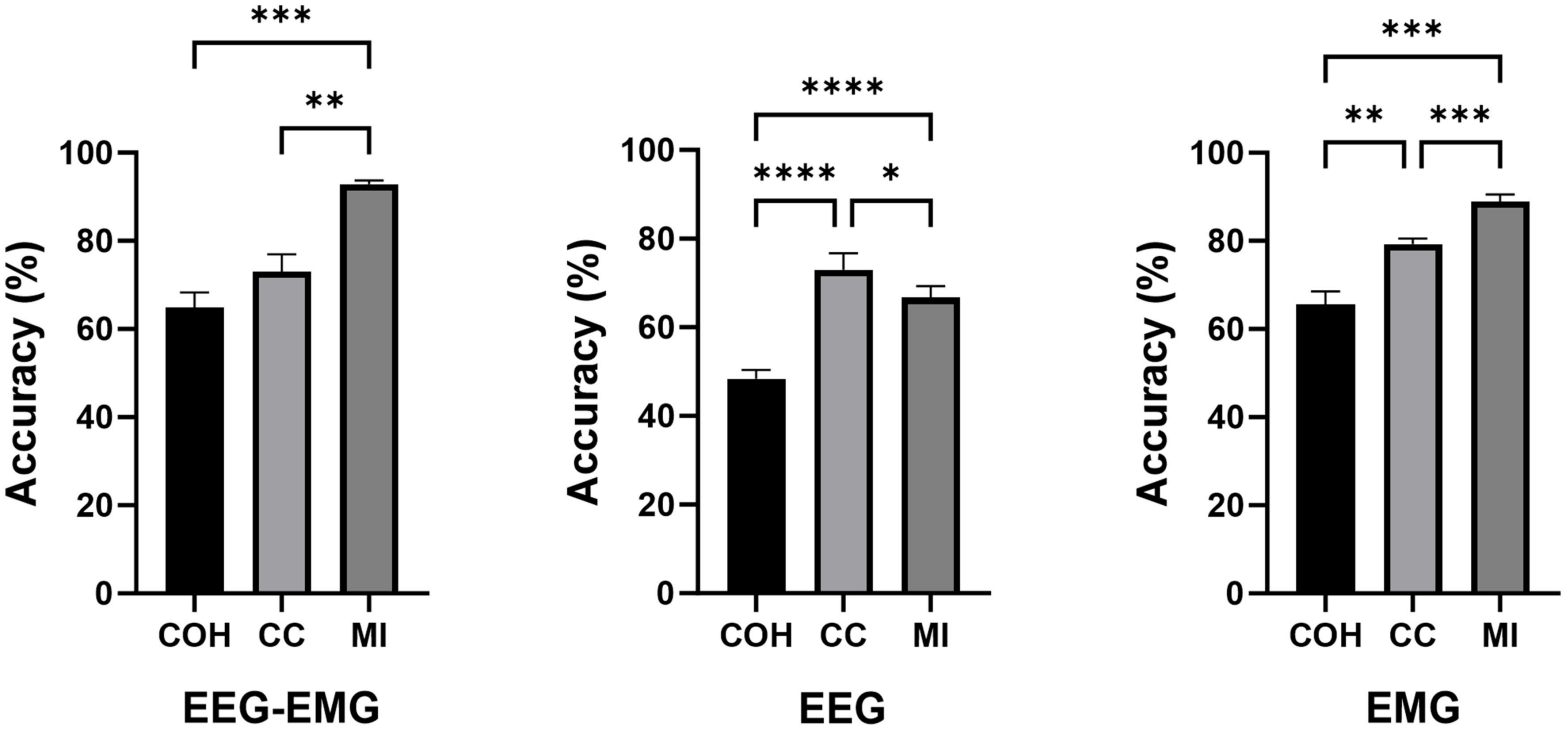
Comparison of classification accuracies across different connectivity methods during the post-fatigue stage (*p < 0.05, **p < 0.01, ***p < 0.001, ****p < 0.0001).

The classification accuracies during the post-fatigue stage are shown in Table 3. Consistent with the pre-fatigue findings, the EEG–EMG network weighted by MI achieved highest accuracy (92.87%). As presented in Figure 5, there were significant differences in classification accuracy among different connectivity methods across all modalities (EEG–EMG: *p* = 0.0001; EEG: *p* = 5.4034e-07; EMG: *p* = 0.0001). For the EEG–EMG network, the accuracies were 64.85, 73.08, and 92.87% for COH, CC and MI, respectively. Post-hoc comparisons analysis showed significant differences between COH and MI (*p* = 0.0001), and CC and MI (*p* = 0.0035). For the EEG network, the accuracies were 48.52, 72.95, and 66.68% for COH, CC, and MI, respectively. Significant differences were found between COH and CC (*p* = 3.5790e-05), COH and MI (*p* = 6.3602e-06), and CC and MI (*p* = 0.0424). For the EMG network, the accuracies were 65.58, 79.32, and 88.94% for COH, CC, and MI, respectively. Significant differences were observed between COH and CC (*p* = 0.0077), COH and MI (*p* = 0.0003), and CC and MI (*p* = 0.0002). The findings indicate that MI consistently remains the most effective indicator, regardless of the fatigue condition, thereby reinforcing its reliability and robustness for intention detection in potentially fatigued stage.

**Table 3:** The classification accuracies of healthy subjects for different functional connectivity methods across three modalities during the post-fatigue stage.

Subjects	Decoding accuracy % (post-fatigue)								
	EEG–EMG			EEG			EMG		
	COH	CC	MI	COH	CC	MI	COH	CC	MI
S1	70.63	69.38	96.88	42.5	69.38	58.13	77.5	78.75	95
S2	62.5	83.13	90.63	55	83.75	77.5	59.38	72.5	81.88
S3	77.5	91.25	95	57.5	90.63	75	71.88	81.25	88.75
S4	72.5	58.13	90	43.75	57.5	56.88	74.38	76.88	84.38
S5	67.5	66.25	91.88	43.75	69.38	61.88	66.25	83.75	89.38
S6	46.25	76.88	90.63	46.88	76.25	70.63	53.13	78.75	86.88
S7	56.88	62.5	94.38	46.63	63.13	66.25	60	81.25	93.13
S8	65	77.14	93.57	52.14	73.57	67.14	62.14	81.43	92.14
Mean	64.85	73.08	92.87	48.52	72.95	66.68	65.58	79.32	88.94
Std	9.81	11.07	2.47	5.65	10.70	7.51	8.42	3.48	4.47

### 3.3 Effects of window size and window range

**Figure 6:**
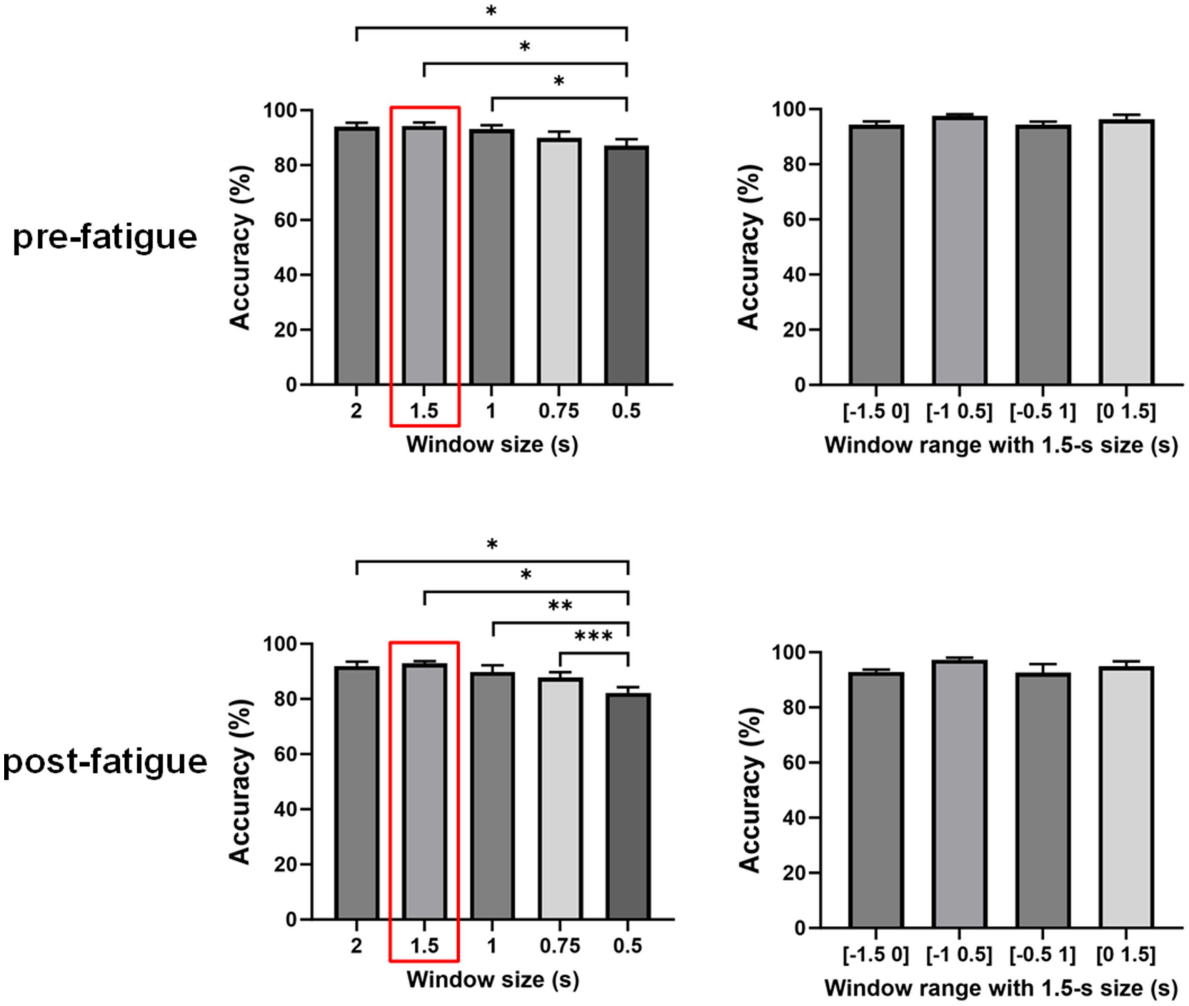
Comparison of classification accuracies across different window sizes and ranges using the multimodal fusion method (*p < 0.05, **p < 0.01, ***p < 0.001).

To further explore the impact of window size on the classification accuracy, we applied this MI-based EEG–EMG fusion method to different window lengths prior to movement execution, specifically 2-s, 1.5-s, 1-s, 0.75-s, and 0.5-s windows. A one-way repeated measures ANOVA was used to evaluate the significance of differences among the window sizes. As shown in Figure 6, the statistical analysis revealed a significant effect of window size on classification accuracy during the both pre- and post-fatigue stages (pre-fatigue: *p* = 0.0041; post-fatigue: *p* = 0.0015). During the pre-fatigue stage, post-hoc comparisons showed that the 2-s, 1.5-s, and 1-s windows achieved significantly higher accuracy than the 0.5-s window (*p* = 0.0362, *p* = 0.0188, *p* = 0.0185, respectively), with the 1.5-s window providing the highest average accuracy among all sizes. During the post-fatigue stage, post-hoc comparisons showed that the 2-s, 1.5-s, 1-s and 0.75-s windows achieved significantly higher accuracy than the 0.5-s window (*p* = 0.0150, *p* = 0.0119, *p* = 0.0072 and p = 0.0003, respectively), with the 1.5-s window also providing the highest average accuracy among all sizes. Therefore, subsequent analyses still focused on the 1.5-s window.

We further investigated the effect of different time windows on classification accuracy by performing a repeated measures one-way ANOVA to compare the decoding performance of four windows: [−1.5, 0] s, [−1, 0.5] s, [−0.5, 1] s, and [0, 1.5] s. As shown in Figure 4, there were no significant differences in decoding accuracy across various time windows (pre-fatigue: *p* = 0.1351; post-fatigue: *p* = 0.2241). This finding suggests that the window [−1.5 0] s is comparable to trans-movement and ongoing-movement windows for detecting sitting and standing intentions.

### 3.4 Comparison of the performance between the multimodal and unimodal methods

**Figure 7:**
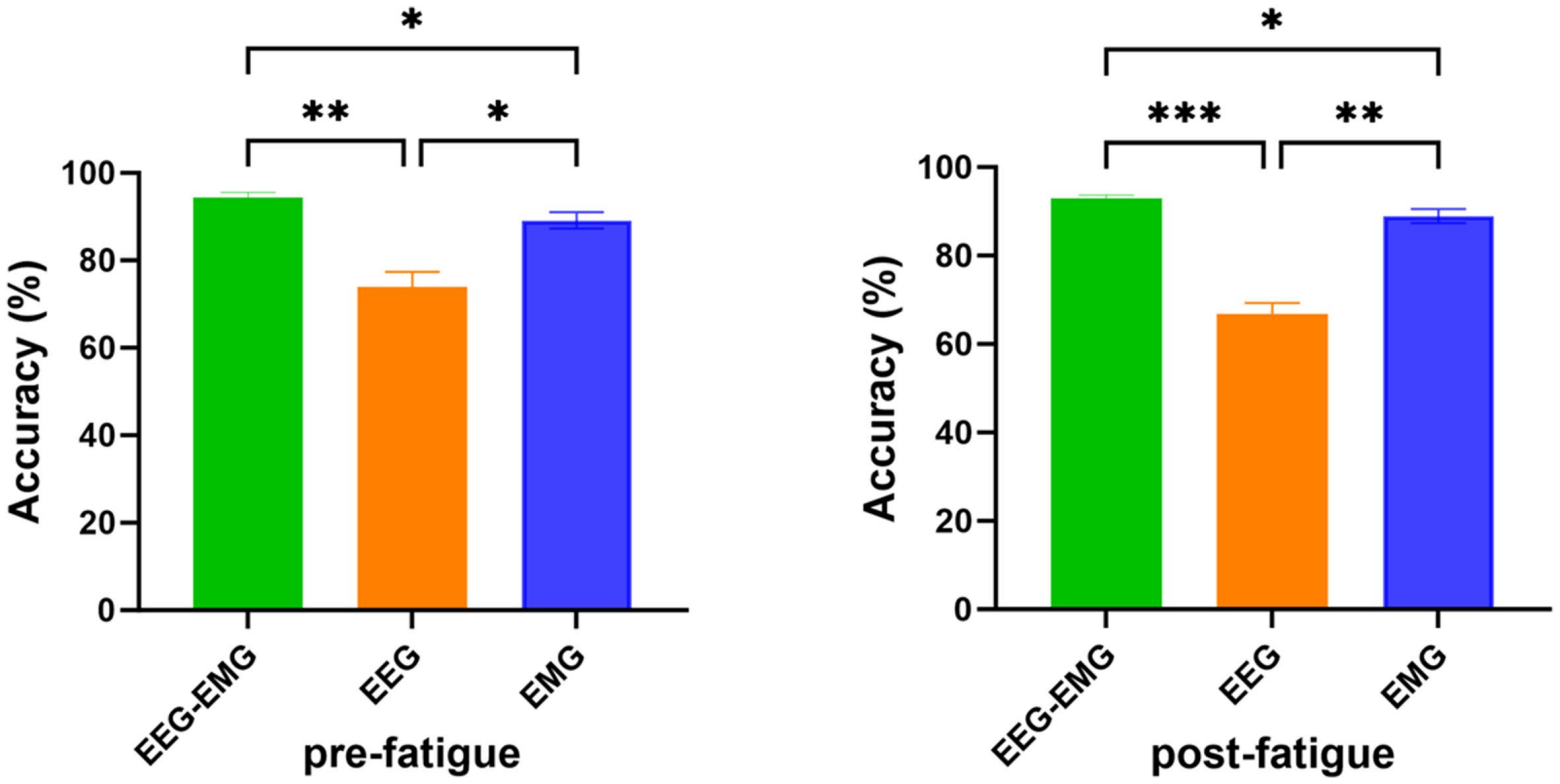
Comparison of classification accuracy of EEG–EMG, EEG and EMG modalities (*p < 0.05, **p < 0.01, ***p < 0.001).

In Figure 7, a one-way repeated measures ANOVA revealed significant differences in classification accuracy among EEG–EMG (pre-fatigue: 94.33 ± 3.48%, post-fatigue: 92.87 ± 2.47%), EEG (pre-fatigue: 73.89 ± 9.87%, post-fatigue: 66.68 ± 7.51%), and EMG (pre-fatigue: 89.16 ± 5.28%, post-fatigue: 88.94 ± 4.47%) modalities during both the pre-fatigue and post-fatigue stages (pre-fatigue: *p* = 0.002; post-fatigue: *p* = 0.0001). Further post-hoc comparisons analysis showed that, during the pre-fatigue stage, EEG–EMG achieved significantly higher accuracy compared to both EEG and EMG (*p* = 0.0032 and *p* = 0.0345, respectively), while EMG outperformed EEG (*p* = 0.0261). During the post-fatigue stage, EEG–EMG maintained significantly higher accuracy than both EEG and EMG (*p* = 0.0001 and *p* = 0.011, respectively), and EMG performed better than EEG (*p* = 0.0011). These findings demonstrated that the EEG–EMG fusion approach significantly improved classification performance compared to unimodal methods, highlighting its robustness and reliability in all conditions.

**Figure 8:**
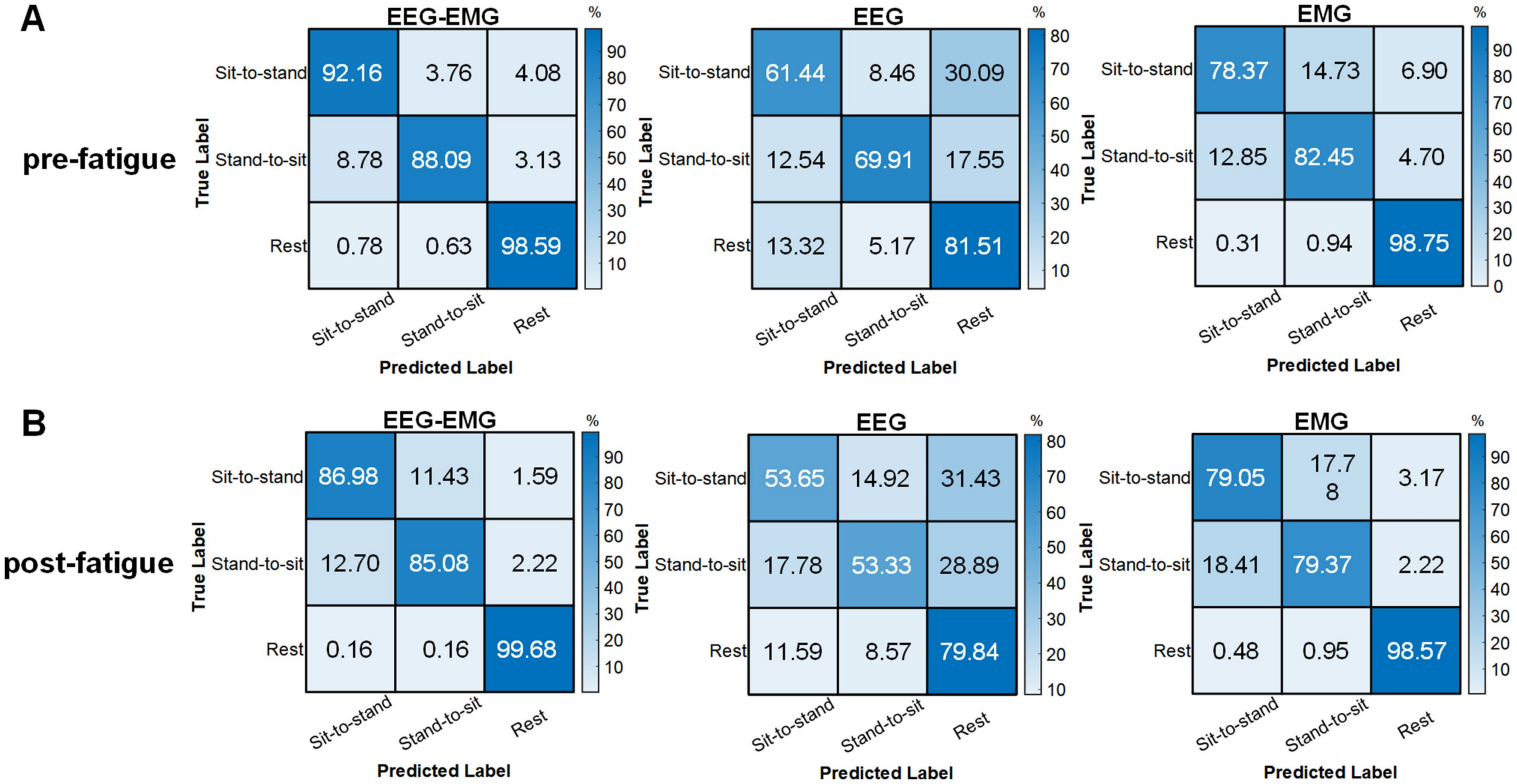
Confusion matrices obtained using the classifier with EEG–EMG, EEG, and EMG during the **(A)** pre-fatigue and **(B)** post-fatigue stages.

The pre-fatigue confusion matrices for each modality are presented in Figure 8A. The EEG–EMG network demonstrated superior accuracy in distinguishing between dynamic movements intentions (sit-to-stand and stand-to-sit), with classification accuracies of 92.16% for sit-to-stand and 88.09% for stand-to-sit, respectively. For the rest state, both the EEG–EMG and EMG networks achieved high classification accuracies (EEG–EMG: 98.59%, EMG: 98.75%). However, the accuracy of EMG for dynamic movements was lower (78.37% for sit-to-stand and 82.45% for stand-to-sit). The EEG network had the lowest classification accuracy across all three classes (sit-to-stand: 61.44%, stand-to-sit: 69.91%, rest: 81.51%). These findings underscore the enhanced performance of the MI-based EEG–EMG network, especially in accurately detecting sitting and standing intentions compared to unimodal networks.

The post-fatigue confusion matrices for each modality are shown in Figure 8B. The EEG–EMG network achieved the highest classification accuracy across all three movement states (sit-to-stand: 86.98%, stand-to-sit: 85.08%, rest: 99.68%). Notably, EEG showed a reduction in its capacity to differentiate between movement types after fatigue; the classification accuracy for sit-to-stand decreased from 61.44% during pre-fatigue to 53.65% during post-fatigue, for stand-to-sit, it dropped from 69.91 to 53.33%, and for rest, it dropped from 81.51 to 79.84%. In contrast, EMG maintained relatively stable performance between the pre-fatigue and post-fatigue stages, with sit-to-stand accuracy marginally increasing from 78.37 to 79.05%, stand-to-sit accuracy decreasing from 82.45 to 79.37%, and rest accuracy slightly decreasing from 98.75 to 98.57%. Nevertheless, the decoding performance based on EMG alone remained lower compared to the EEG–EMG based method.

We further compared the decoding performance between the pre-fatigue and post-fatigue stages. There was no significant difference in decoding accuracy between the pre-fatigue and post-fatigue conditions for the EEG–EMG network weighted by MI (*p* = 0.3226, paired *t*-test). Overall, EEG–EMG demonstrated superior robustness and maintained high accuracy in detecting movement intentions compared to unimodal EEG and EMG, even under fatigued conditions.

### 3.5 SCI patient results

**Figure 9:**
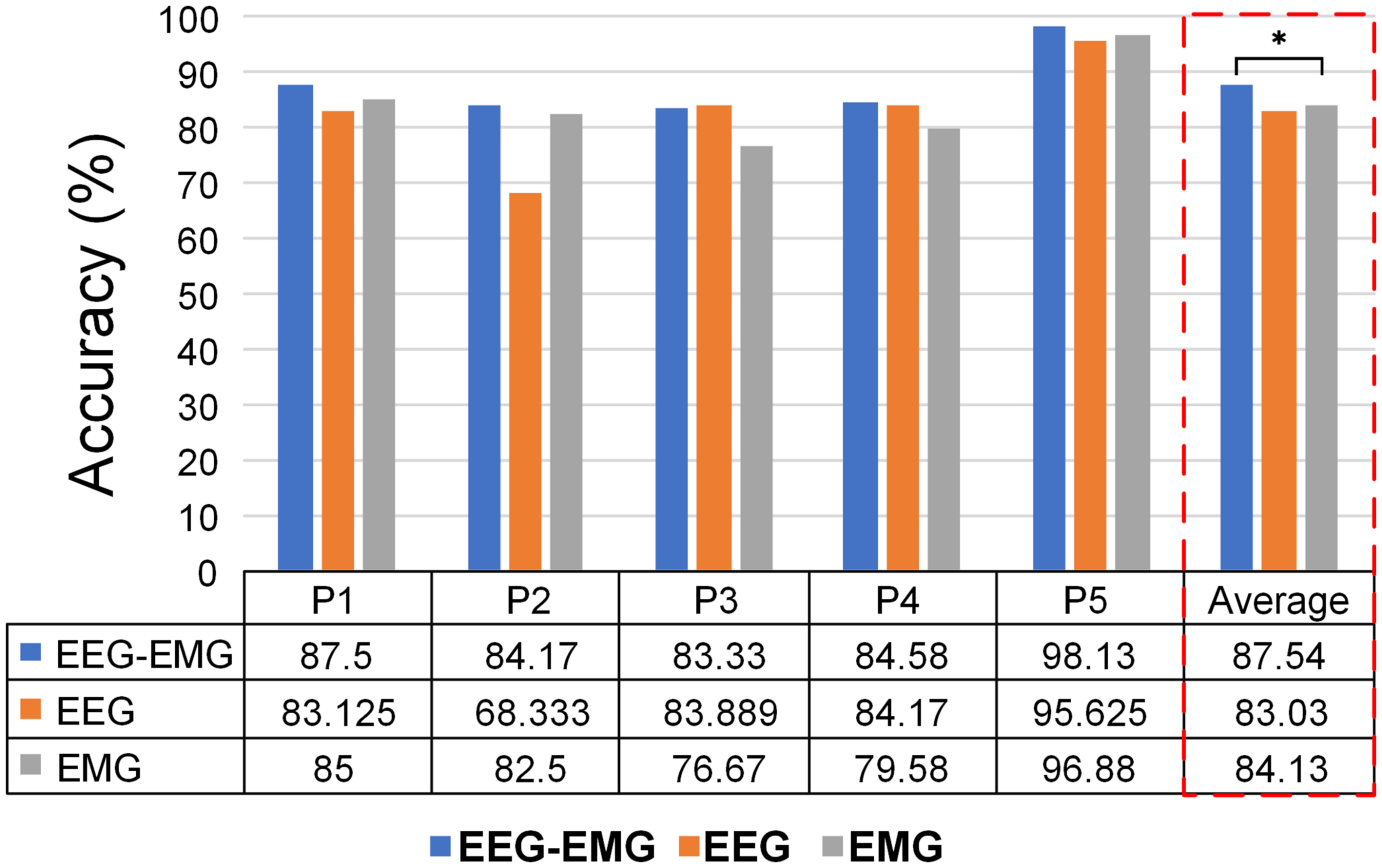
Classification accuracies of different modalities for individual SCI patients.

Furthermore, we performed preliminary testing on five SCI patients to assess the feasibility of the proposed method in real-world rehabilitation applications. Figure 9 displays the intention detection results for each patient. The average accuracy of EEG–EMG fusion approach was 87.54% ± 6.12%, demonstrating high accuracy in intention detection. In comparison, the accuracy of using EMG alone for intention detection was 84.13% ± 7.78%, and the accuracy of using EEG alone is 83.03% ± 9.7%, both showing a decline relative to the fused modality. The improvement achieved by the multimodal fusion approach over the unimodal EMG method was statistically significant (*p* = 0.0303, paired t-test). Although the multimodal approach did not show a statistically significant difference compared to the unimodal EEG method, it provided improvements for most patients. Notably, for P2, the EEG–EMG approach achieved an accuracy increase of 15.84% compared to EEG alone (84.17% vs. 68.33%).

**Figure 10:**
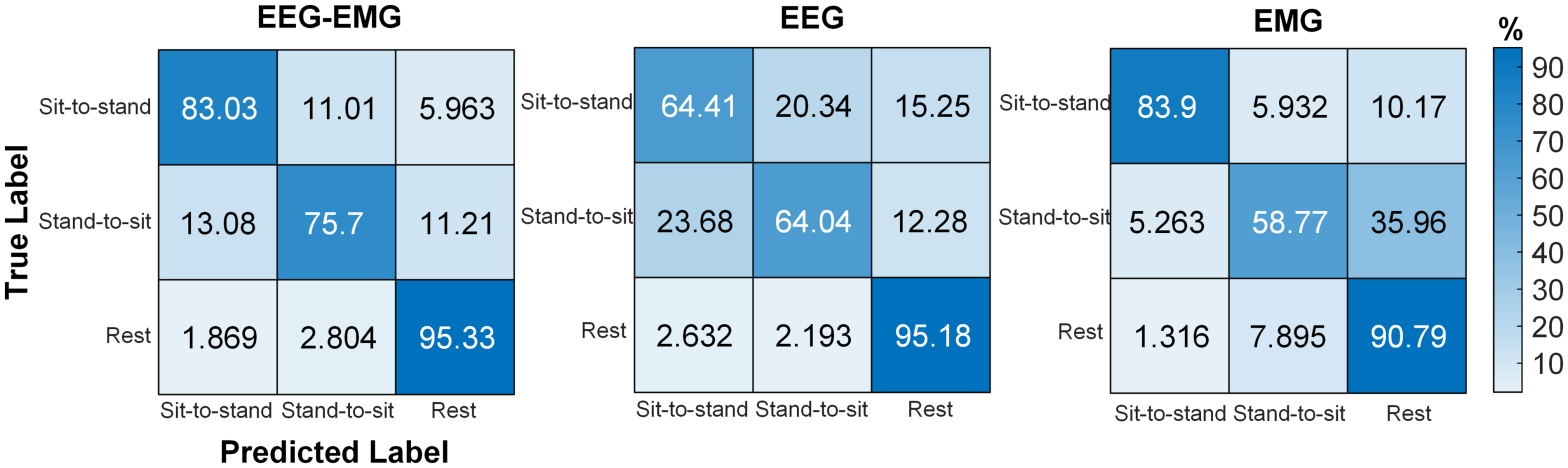
Confusion matrices obtained using the classifier with EEG–EMG, EEG, and EMG modalities for SCI patients.

The averaged confusion matrix for the five SCI patients is shown in Figure 10. Similar to healthy subjects, EEG–EMG also showed higher performance in differentiating between sit-to-stand and stand-to-sit (sit-to-stand: 83.03%, stand-to-sit: 75.7%), compared to EEG (sit-to-stand: 64.41%, stand-to-sit: 64.04%). Although EMG achieved an accuracy of 83.9% for sit-to-stand, it was only 58.77% for stand-to-sit, with 35.96% of “Stand-to-sit” were misclassified as “Rest.” EEG–EMG consistently achieved high accuracy in detecting “Rest” (95.33%). However, EMG showed a reduction in its capacity to detect “Rest” for SCI patients (90.79%), compared to healthy subjects (98.75%). In contrast, EEG successfully classified “Rest” at a much higher accuracy for the SCI patients (95.18%), compared to the healthy subjects (81.51%). These results indicated that the EEG–EMG fusion approach provided a more robust solution for SCI patients than EEG and EMG alone.

## 4 Discussion

HMI are widely used for the active control of rehabilitation devices. A crucial aspect of designing and implementing effective rehabilitation systems is the ability to predict movement intentions. Early intention recognition allowing time to trigger external devices that provide relevant somatosensory feedback to induce Hebbian plasticity (Grosse-Wentrup et al., 2011). In this study, we proposed and validated a multimodal HMI technology that detects the intention of sitting and standing prior to movement onset based on EEG–EMG network weighted by MI. The experiment results in healthy subjects demonstrated that the proposed fusion method achieved significantly higher accuracy than EEG and EMG alone, and maintained superior performance even after fatigue, highlighting the advantages of integrating multimodal signals for detecting movement intentions. Furthermore, preliminary experiments in SCI patients demonstrated the feasibility of our method, with the proposed fusion method achieving optimal results for most patient. These findings underscore the effectiveness and adaptability of multimodal signals in rehabilitation applications.

**Figure 11:**
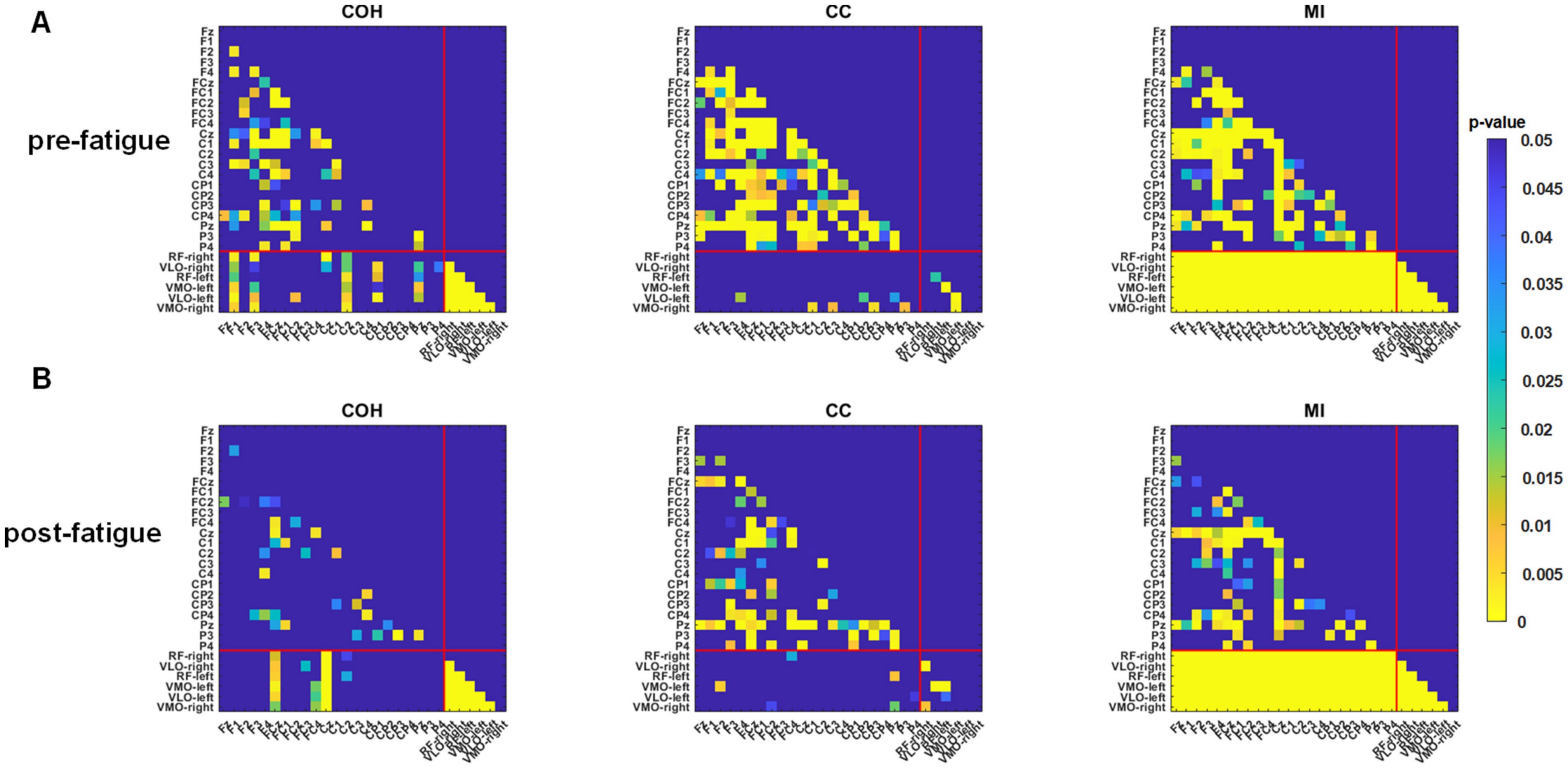
Detailed significant *p*-values for the functional connectivity strength between each pair of channels across three tasks for S5 during the **(A)** pre-fatigue and **(B)** post-fatigue stages.

This study investigated three functional connectivity methods for constructing networks within and between EEG and EMG for movement intention recognition. Our results indicate that the MI-based EEG–EMG networks achieved better classification accuracy compared to those constructed using COH and CC. Unlike COH and CC, which primarily measure linear tendencies, MI assesses both linear and nonlinear statistical dependencies between two time series (Kvalseth, 1987). Given that the human nervous system exhibits complex nonlinear behavior, ranging from the single-neuron level to the system level, emphasizing the necessity of nonlinear analysis in precisely investigating neuronal processing and signal transfer (He and Yang, 2021). These results suggest that MI is better suited for capturing intention-related features between complex neurophysiological signals. To further explore why the EEG–EMG networks weighted by MI outperformed the others, we conducted a Kruskal-Wallis statistical test to identify channel pairs with significant differences across the sit-to-stand, stand-to-sit and rest tasks. In Figure 11A, the results of S5 during pre-fatigue are shown, where each cell in the heatmaps represents the *p*-value of the functional connectivity strength between two channels across different tasks. The analysis revealed that the MI-based EEG–EMG network exhibited a broader distribution of significant channel pairs, especially between EEG and EMG channels, compared with networks weighted by COH and CC. During post-fatigue, as shown in Figure 11B, the interaction and coordination between the brain and muscles remain significant across different tasks. This may explain why EEG–EMG networks can considerably improve the recognition accuracy. It has been shown that the presence of motor intention during upper limb movement alters functional integration between the brain and muscles (Kim et al., 2017). This also suggests that MI is more effective in capturing the nonlinear dependencies between EEG and EMG signals, which are essential for decoding intentions in lower-limb accurately.

We conducted a comparison with several existing EEG–EMG fusion methods for classification to evaluate the performance of our proposed approach. Table 4 presents the comparative experimental results, which consistently demonstrate that the proposed method achieves superior accuracy across the majority of participants.

**Table 4:** Comparison of the classification accuracy (%) between the proposed method and the existing EEG–EMG fusion methods.

Paper	S1	S2	S3	S4	S5	S6	S7	S8	Mean ± Std (%)
Chowdhury et al. (2019)	82.5	71.88	71.88	81.88	75.63	65	80.63	81.64	76.38 ± 6.37
Tryon and Trejos (2020)	93.13	90.63	90	93.75	90.63	90.63	91.25	90	91.25 ± 1.42
Al-Quraishi et al. (2021)	67.5	77.5	68.75	85.63	73.75	67.5	65	76.9	72.82 ± 6.95
Tryon and Trejos (2021)	**97.53**	93.83	95.06	95.06	93.83	**95.06**	87.65	89.74	93.47 ± 3.21
Jo et al. (2022)	78.13	80.63	80.63	79.38	67.5	74.38	76.88	73.08	76.32 ± 4.50
López-Larraz et al. (2024)	81.25	90.63	86.25	78.75	84.38	82.5	75	77.69	82.06 ± 5.03
Proposed	95	**97.5**	**96.25**	**95.63**	**96.25**	86.88	**95.63**	**91.53**	94.33 ± 3.48

The highest value for each subject is indicated in bold.

Detecting movement intentions before the actual onset of movement is crucial for designing and implementing rehabilitation devices with potential real-life applications. The earlier the movement intention can be recognized, the earlier the rehabilitation system can adjust parameters and provide timely activation according to the subjects’ needs. The detection of pre-movement intention with the EEG signal alone is not surprisingly. However, few studies have explored the integration of EEG and EMG signals for intention detection before movement onset. Previous studies have suggested that interactions between EEG and EMG activities begin even before the physical execution of movement (Deng et al., 2023). A possible reason is that in preparation for the movement initiation, the cortical sensory control system begins to deploy attention, which leads to a synchronized oscillations of neuromuscular motor neurons (Zhu et al., 2022). In addition, our results showed that the classification performance using pre-movement windows was similar as using either trans-onset or post-onset windows. Therefore, the proposed method could be a promising tool in close-loop HMI control system.

There is a large body of research demonstrating that movement intention can be decoded from EEG signals on SCI patients (Rohm et al., 2013; King et al., 2015; López-Larraz et al., 2016). It has been demonstrated that substantial neural control information can be extracted from the upper limb muscles of SCI patients (Liu et al., 2014). Our study provides additional evidence that the residual EMG signal from lower limb in SCI patients can also be successfully used to decode movement intention for sitting and standing. Additionally, EEG offers complementary discriminative information, enhancing the classification accuracy. Although the cortex-muscle interactions have been shown to effectively evaluate the residual integrity of the neuromuscular system in SCI patients (Cremoux et al., 2017), little investigation has explored the feasibility of using EEG–EMG functional connectivity for decoding movement intentions. Similar to the healthy subjects, our results show that the EEG–EMG network weighted by MI also achieved superior decoding performance in SCI patients, compared to individual EEG and EMG networks. It is worth noting that when using the EEG network weighted by MI, the average classification accuracy of the SCI patients was higher than that of the healthy subjects. This could be attributed to the fact that, SCI patients with lower limb functional impairments, had to exert more effort and concentration to perform sitting and standing, which are complex tasks. Furthermore, we found considerable number of stand-to-sit intentions were misclassified as rest. This can be attributed to the fact that the stand-to-sit movement involves eccentric muscle contractions to control the descent, which is more complex than concentric muscle contractions (Latella et al., 2019). In SCI patients, the control of eccentric contractions can be impaired due to significant reductions in muscle strength, coordination and functionality (Balbinot et al., 2021). Additionally, although studies have shown that SCI patients generates greater activation during eccentric contractions (Souza et al., 2005), the increased activation which may be inconsistent due to muscle weakness, could contribute to difficulties in distinguishing stand-to-sit intentions from rest using EMG data alone.

A limitation of the proposed method described in this work is the choice of functional connectivity measures. Several other methods have also been used to analyze functional connectivity in neuroscience, such as maximal information coefficient (Reshef et al., 2011), wavelet coherence (Xi et al., 2021), cross-spectral coherence (Yang et al., 2016). It would be worthwhile to explore different connectivity methods in future studies to further improve the performance of intention detection accuracy based on EEG–EMG networks. Additionally, our study is limited by the sample size and the inclusion of SCI patients who have residual EMG activity. The effect of spinal cord injury on motor ability is diverse, depending on the location of the injury and the severity of the injury. There has been no consistent conclusion on how the characteristics of EMG change following spinal cord injury. Meanwhile, SCI also alters brain activity related to movement (Castro et al., 2013). The heterogeneity of SCI may result in differences in the neural reorganization processes and muscle strength of patients (Freund et al., 2013). To verify the robustness and generality of the fusion method, future studies should involve more diverse patient populations, encompassing various subtypes of SCI. Furthermore, it remains to be explored whether the proposed method can be generalized to other functional lower limb movements, such as gait initiation, step up, back step, which are relevant to activities of daily living.

## 5 Conclusion

In this research, we introduced a multimodal HMI that integrates EEG and EMG signals to improve the accuracy and stability of movement intention detection of sitting and standing by learning discriminative spatial network topology patterns, which is crucial for controlling rehabilitation systems timely and inducing motor learning and Hebbian-associated plasticity. The feasibility of the proposed method was validated through experiments with eight healthy subjects and five SCI patients with residual EMG signals. The fusion method significantly improves classification accuracy compared with the results obtained from single modality data. Our results showed that functional connectivity measures play a crucial role in recognition performance, with the MI-based EEG–EMG network significantly outperforming COH and CC-based methods. The average classification accuracy was 94.33 and 92.87% for healthy subjects at the pre-fatigue stage and the post-fatigue stage, respectively. For SCI patients, the average classification accuracy is 87.54%, highlighting its potential reliability in real-world clinical rehabilitation settings. Furthermore, the classification performance using pre-movement window was comparable with the trans-onset or post-onset windows. The proposed approach offers a promising tool for developing a closed-loop HMI neurorehabilitation system.

## Data Availability

The raw data supporting the conclusions of this article will be made available by the authors, without undue reservation.
