# Supplementary material for: Fusion of EEG and EMG signals for detecting pre-movement intention of sitting and standing in healthy individuals and patients with spinal cord injury
Source: Front Neurosci. 2025 Jan 24;19:1532099. doi: 10.3389/fnins.2025.1532099 (PMC11802573; doi:10.3389/fnins.2025.1532099)
Supplement: Supplementary file 1 [file Data_Sheet_1.pdf]

Supplementary Material

S1

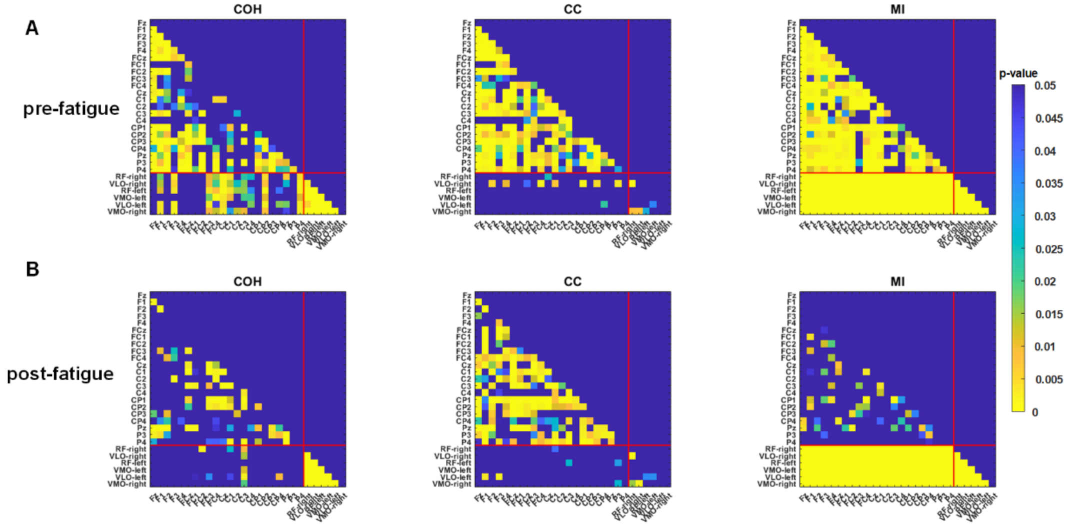

Supplementary Figure 1. Detailed significant p-values for the functional connectivity strength between each pair of channels across three tasks for S1 during the (A) pre-fatigue and (B) post-fatigue stages.

S2

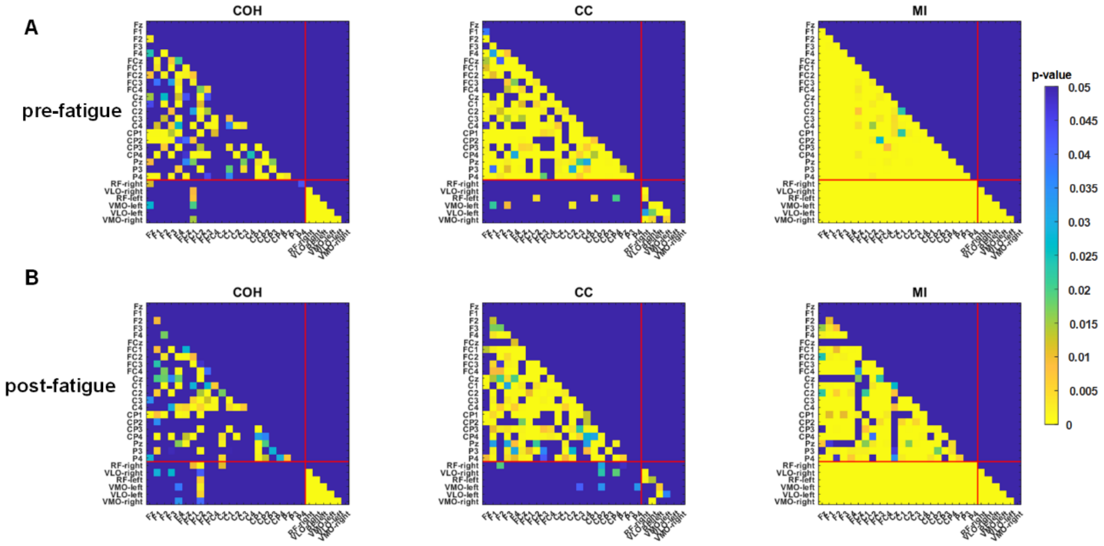

Supplementary Figure 2. Detailed significant p-values for the functional connectivity strength between each pair of channels across three tasks for S2 during the (A) pre-fatigue and (B) post-fatigue stages.

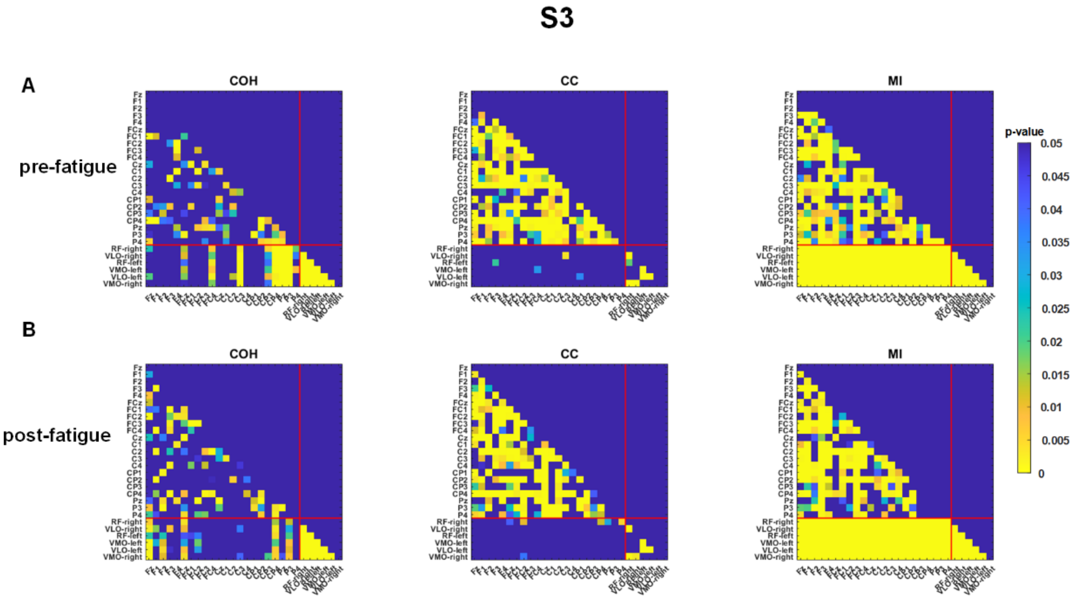

Supplementary Figure 3. Detailed significant p-values for the functional connectivity strength between each pair of channels across three tasks for S3 during the (A) pre-fatigue and (B) post-fatigue stages.

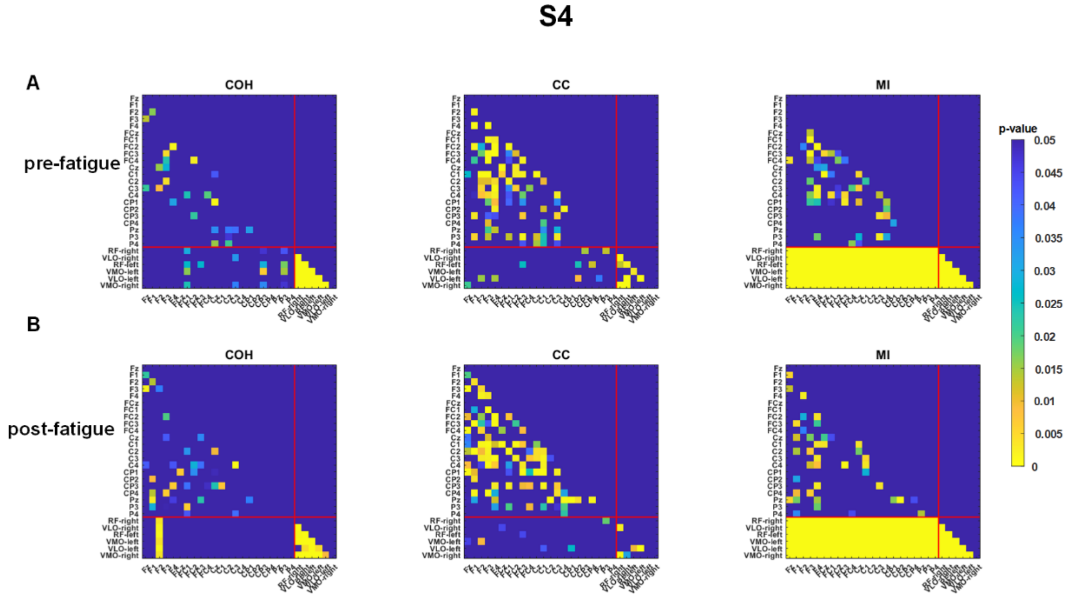

Supplementary Figure 4. Detailed significant p-values for the functional connectivity strength between each pair of channels across three tasks for S4 during the (A) pre-fatigue and (B) post-fatigue stages.

S6

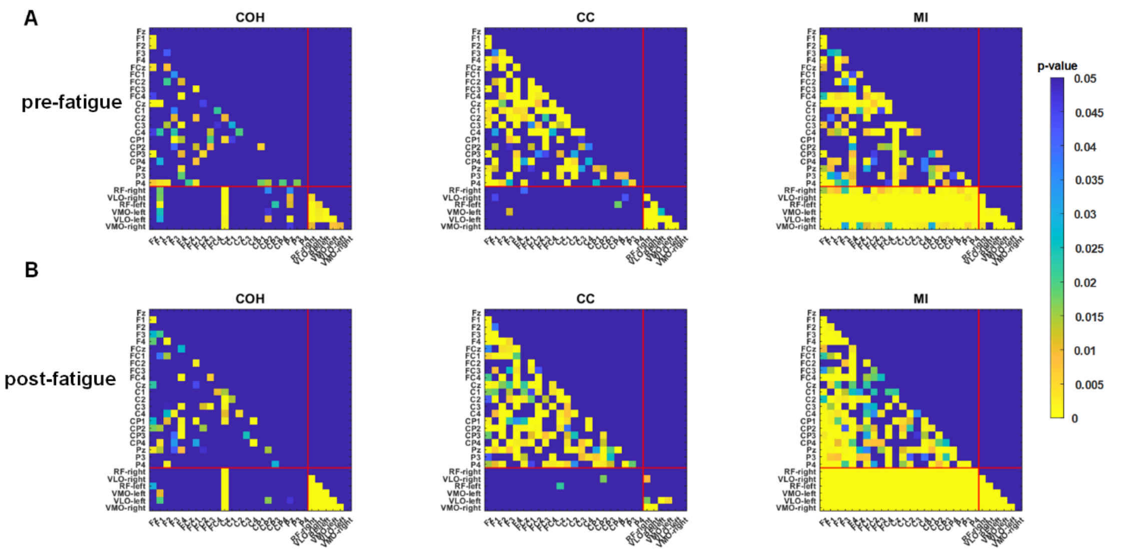

Supplementary Figure 5. Detailed significant p-values for the functional connectivity strength between each pair of channels across three tasks for S6 during the (A) pre-fatigue and (B) post-fatigue stages.

S7

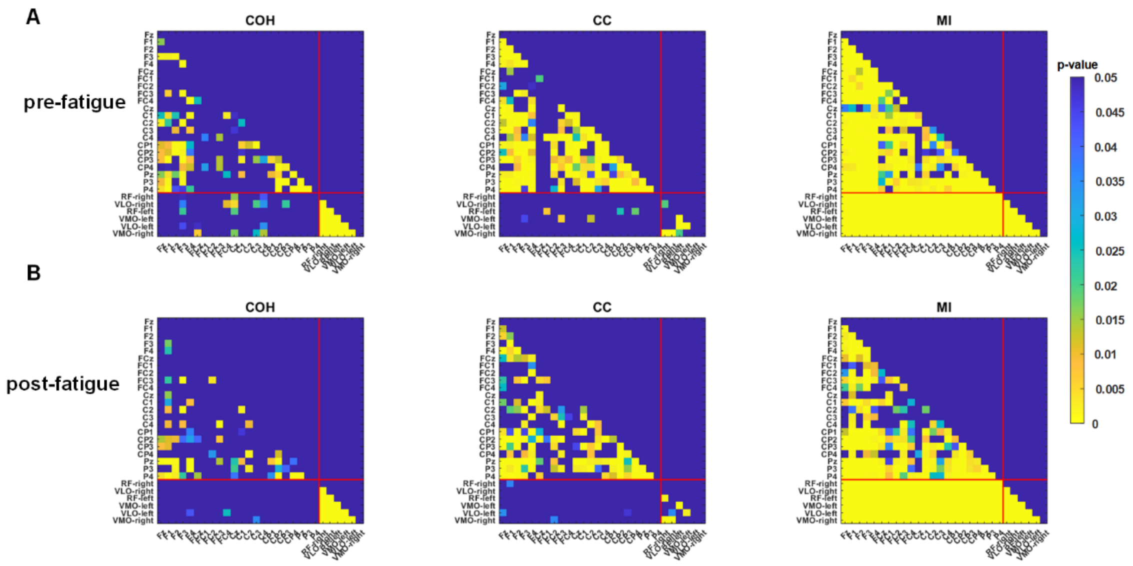

Supplementary Figure 6. Detailed significant p-values for the functional connectivity strength between each pair of channels across three tasks for S7 during the (A) pre-fatigue and (B) post-fatigue stages.

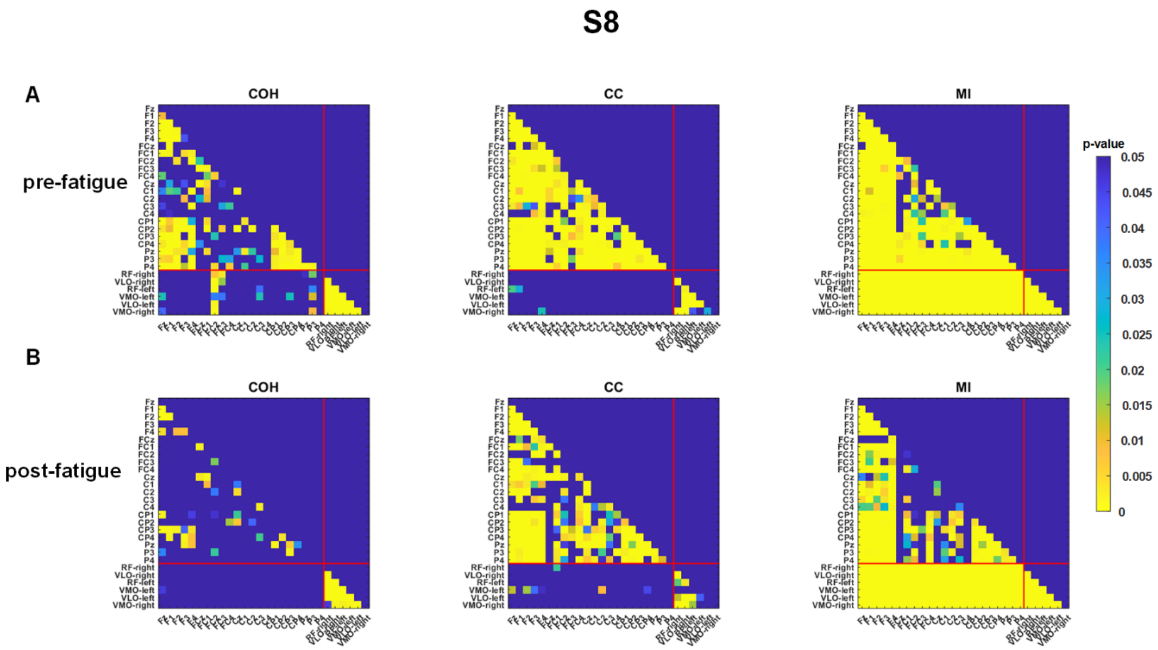

Supplementary Figure 7. Detailed significant p-values for the functional connectivity strength between each pair of channels across three tasks for S8 during the (A) pre-fatigue and (B) post-fatigue stages.
